# SCC: an accurate imputation method for scRNA-seq dropouts based on a mixture model

**DOI:** 10.1186/s12859-020-03878-8

**Published:** 2021-01-06

**Authors:** Yan Zheng, Yuanke Zhong, Jialu Hu, Xuequn Shang

**Affiliations:** School of Computer Science, Northwestern Polytechnical University, West Youyi Road 127, Xi’an, 710072 China

**Keywords:** ScRNA-seq, Noise, Mixture model, Dropouts identification, Gene expression estimation

## Abstract

**Background:**

Single-cell RNA sequencing (scRNA-seq) enables the possibility of many in-depth transcriptomic analyses at a single-cell resolution. It’s already widely used for exploring the dynamic development process of life, studying the gene regulation mechanism, and discovering new cell types. However, the low RNA capture rate, which cause highly sparse expression with dropout, makes it difficult to do downstream analyses.

**Results:**

We propose a new method SCC to impute the dropouts of scRNA-seq data. Experiment results show that SCC gives competitive results compared to two existing methods while showing superiority in reducing the intra-class distance of cells and improving the clustering accuracy in both simulation and real data.

**Conclusions:**

SCC is an effective tool to resolve the dropout noise in scRNA-seq data. The code is freely accessible at https://github.com/nwpuzhengyan/SCC.

## Background

Advances in gene sequencing technology have made genome research more and more popular in the past decades [1]. Although methods based on bulk RNA-seq can obtain the genome-wide RNA sequence expression information, the resulting gene expression profiles are only the average values of the different cell types, which cause the studies of gene expression limited to the analysis of pooled populations of cells. The heterogeneity in cells is neglected and mutations present only in a few cells are substantially hidden (such as early cancer cells) [2]. The analysis of cell clusters does not show cell heterogeneity, which is also an important feature of organ development [3]. In the process of organ development, the progenitor cells undergo diverse differentiation decisions to become specific cell types. Therefore, technology that define the gene expression of individual cells is necessary for a better understand of the differentiation and heterogeneity of cells.

Single-cell RNA sequencing (scRNA-seq), which enables rapid determination of the precise gene expression patterns of tens of thousands of individual cells has been propposed. [1]. scRNA-seq is vital for exploring the dynamic development process of life and studying the regulation mechanism of genes, which also can be used to discover new cell types. However, there are still some limitations for scRNA-seq technology, the key limitation is the noise of the scRNA-seq data, which is mainly caused by the poor sensitivity of scRNA-seq technology [4]. Current scRNA-seq technology can detect only about 10 percent of the mRNA molecules that are actually present. Therefore, low-expression genes are difficult to detect in scRNA-seq data [3]. In addition, the expression of genes is not in a steady-state manner in different periods. Batch effect can also cause noise. It is estimated that there are eighty percent noise is caused by technical limitations and the remaining twenty percent is estimated to be of biological origin. The primary challenge in the scRNA-seq data analysis is how to remove the noise of data.

**Fig. 1:**
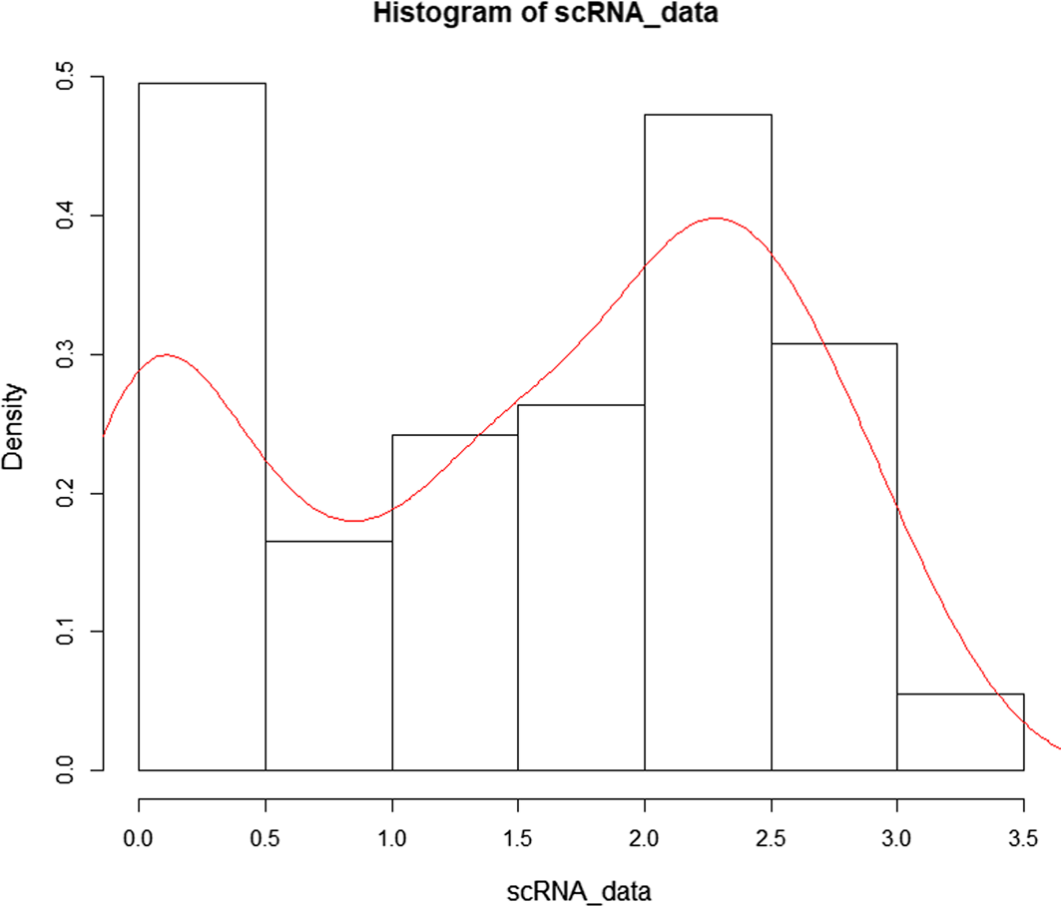
The bimodal expression distribution. The typical bimodal expression distribution of scRNA-seq data (The eleventh gene in mECS, which is a dataset about mammary epithelial cells). The detection rate of mRNA is 5–15%. The genes with low expression levels are difficult to detect. Therefore, the expression of scRNA-seq data is either strongly zero or high expression. We call this distribution a bimodal distribution

As shown in Fig. 1, the scRNA-seq data tend to be bimodal expression distribution [5]. Although many zero counts in scRNA-seq data are true absence of expression, a big part is caused by technical factors. There are many approaches for solving the noise of scRNA-seq data. scImpute estimates the true expression of genes through clustering similar cells and SAVER recovers the true expression levels of genes by a method that takes advantage of gene-to-gene relationships [6, 7]. Because of the poor sensitivity of scRNA-seq technology, the scRNA-seq data is incomplete. The detection rate of mRNA is only 5-15 percent, so genes with low expression levels are difficult to detect [1]. Although it is hard to get the complete data for low expression genes, if we detect mRNA in multiple cells with the same type, low expression genes are likely to be detected in a small fraction of cells. Once the cells with the same cell types are clustered, we can combine all gene expression data from the same type cells to impute complete gene expression data [7]. Therefore, the main idea of our method is to obtain more complete gene expression data by integrating gene expression data of similar cells, which is similar to scImpute. However, scImpute may remove the cell-to-cell heterogeneity because scImpute impute scRNA-seq data by clustering all cells with the same types while cell-to-cell heterogeneity is also of great significance for exploring cell heterogeneity. In addition, clustering cells into true types is very difficult.

In fact, in scRNA-seq, even in the same cell type, cells with different volumes still have very different mRNA transcript number [5]. Therefore, even if scImpute can clustering cells into true types, the heterogeneity in the same cell type will be neglected while intra cluster heterogeneity also plays an important role in subsequent analysis. In a word, scImpute is a method of clustering similar cells first and then imputing gene expression data, which obtain complete data by clustering, lead to over smoothing of the gene expression data and neglection of intra cluster heterogeneity. Therefore, an accurate expression recovery method that can preserve heterogeneity is essential. In addition, the volume is an important factor that should be considered because it affects gene expression in scRNA-seq. Different cell volumes lead to different mRNA transcript numbers, which leads to different mRNA capture numbers. Methods like scImpute and SAVER ignore this factor. In this paper, we propose a new method named scRNA-seq complementation (SCC), which can modify the data of scRNA-seq and reduce the intra-class distance of cells. In SCC, we replace clustering similar cells with finding the nearest neighbor cells of each cell. In this way SCC can not only obtain the complete gene expression data but also preserve cell-to-cell heterogeneity.

**Fig. 2:**
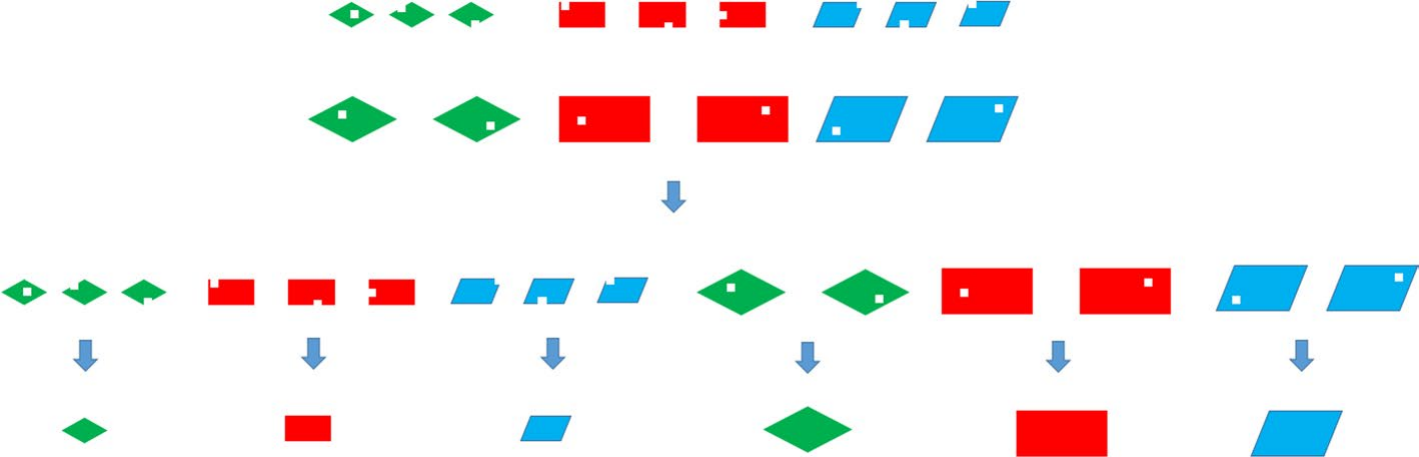
The complementation of scRNA-seq data. A quadrilateral represents a cell and the different colors represent different cell types and different sizes represent different cell volumes. The data of scRNA-seq is incomplete and the holes in quadrilaterals represent the missing information. Although the data of each cell is incomplete, we can still bring together similar cells. We obtain complete cell data by complementing similar cells

The main idea of SCC is shown in Fig. 2. In Fig. 2, there are three different cell types represented by different colors and shapes. The sizes of quadrilaterals represent different cell volumes and the holes in quadrilaterals represent the dropouts in scRNA-seq data. In order to solve the dropouts of scRNA-seq data, we cluster similar cells and obtain the complete cell data by complementation of similar cells. In SCC, for every cell, we find the nearest neighbor cells with similar volume and modify the dropouts of the cell by the complement of neighbor cells. Compared with scImpute, we retain the cell-to-cell Heterogeneity. The result shows that our method can reduce the intra-class distance of cells and enhance the clustering of cell subpopulation.

## Results

SCC does not need the cell type as prior information and the scRNA-seq matrix is the only input. The output of SCC is a modified scRNA-seq matrix. Besides, SCC is memory-efficient because it only modifies one cell at a time. We apply SCC to one simulation dataset and three real scRNA-seq datasets (Kolod, Pollen and Usoskin). The result shows that SCC can significantly reduce the intra-class distance of cells and enhance the clustering of cell subpopulation.

### The simulate data

**Fig. 3:**
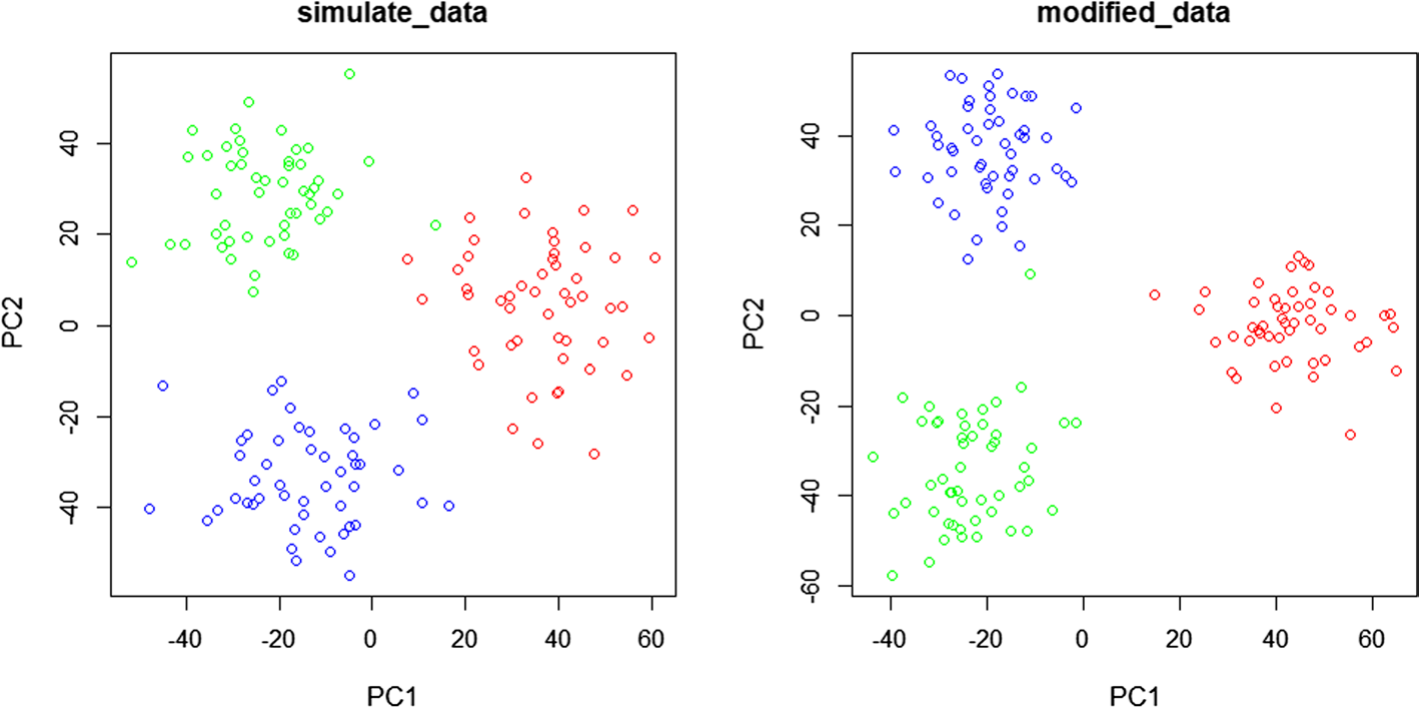
The visualization of simulate data. We run SCC in the simulate dataset. The left chart is the raw simulate data and the right data is the simulate data after modification

We use the scSimulator function to create the simulate data. The simulate data contains 3 cell types, 150 cells and 8180 genes. For the simulate data, we get the modified data by SCC and visualize the raw data and modified data by PCA. The visualization is shown in Fig. 3. The left part is the raw simulate data and right part is the modified data. As shown in Fig. 3, the modified data is more intensive. The Adjusted Rand Index (ARI) can be used to calculate the similarity between the clustering trsult and real types [8]. The range of ARI values is between $-\,1$ and 1. The negative value means that the clustering result is bad, indicating that the labels are independently distributed. The values of good clustering results are positive (1 is the best result), indicating that the distribution of the two labels is identical. We use K-means to cluster the cell and calculate the ARI values of raw data and modified data. The ARI values are 0.4394281 and 0.5233112 respectively. The modified data result is better than the raw data. So we can conclude that SCC is an effective method to recover gene expression.

### The real data

We alse run SCC in the real datasets (Kolod, Pollen and Usoskin). Kolod (704 cells, 13,473 genes) is a dataset about pluripotent cells under different environmental conditions and Pollen (249 cells, 6982 genes) is a dataset contains eleven cell populations including neural cells and blood cells. Usoskin (622 cells, 17,772 genes) is a neuronal cell dataset with sensory subtypes.

#### SCC can resolve the dropouts in scRNA-seq

We counted the number of zero values in the GSE76381 (Adult), which is downloaded from NCBI website. The num of genes is 18,219 and the num of cells is 243. In 4,427,217 gene expression values, 3,429,466 gene expression values are zeros. Zero values account for 77.46% of the total gene expression values. After the modification of gene expression, the ratio of zero values dropped to 68%. 418,815 dropouts are resolved and the rest 3,010,651 zero expression value are identified as true zero expression. Therefore, we can draw a conclusion that SCC can assign value to dropouts and retain a part of real zero values.

#### SCC can reduce the the intra-class distance of cells

We use intra-class distance and inter-class distance to evaluate the performance of SCC modification. The intra-class distance is the mean square distance between sample cells of the same cell types and the inter-class distance is the mean square distance of the sample cells of the different cell types. The smaller the intra-class distance is, the better the modification result is. The smaller the inter-class distance is, the worse the modification result is [9]. We hope that the intra-class distance of cells is smaller and the inter-class distance of cells is larger. We assume that the $K_{i}$ is the $K_{i}$ class, $N_{i}$ is the number of cell in the $K_{i}$ class and $X^{i}_{k}$ is the kth cell value in the $K_{i}$ class (Eq. 1):

1 $$\begin{aligned} &Dis_{intra}=\sum _{K_{i}} \frac{1}{N_{i}*N_{i}} \sum _{k=1}^{N_{i}} \sum _{l=1}^{N_{i}} Dis(X^{i}_{k},X^{i}_{l})\\&Dis_{inter}=\sum _{K_{i}} \sum _{K_{j}} \frac{1}{N_{i}*N_{j}} \sum _{k=1}^{N_{i}} \sum _{l=1}^{N_{j}} Dis(X^{i}_{k},X^{j}_{l}) \end{aligned}$$

After we obtain the matrix of modification, we calculate the intra-class distance and inter-class distance by the formulas above. However, the two distances are used to measure the performance of the result is not convenient. We use another value $Dis=Dis_{intra}/Dis_{inter}$ to describe the performance of result. For the new distance *Dis*, the smaller the value is, the better the performance of the result is. We run SCC, scImpute and SAVER in three different public scRNA-seq datasets and calculate the *Dis* (The three datasets are Kolod, Pollen and Usoskin). Compared with other methods, SCC can significantly reduce the intra-class distance of cells. The result as shown in Table 1.

**Table 1:** Table of cell distance

	Raw data	SCC	scImpute	SAVER
Kolod	27,272.63	21,138.75	27,193.77	15,774.53
Pollen	157.074	118.77	561.21	167.564
Usoskin	21,467.77	19,498.95	23,502.52	20,341.59

**Fig. 4:**
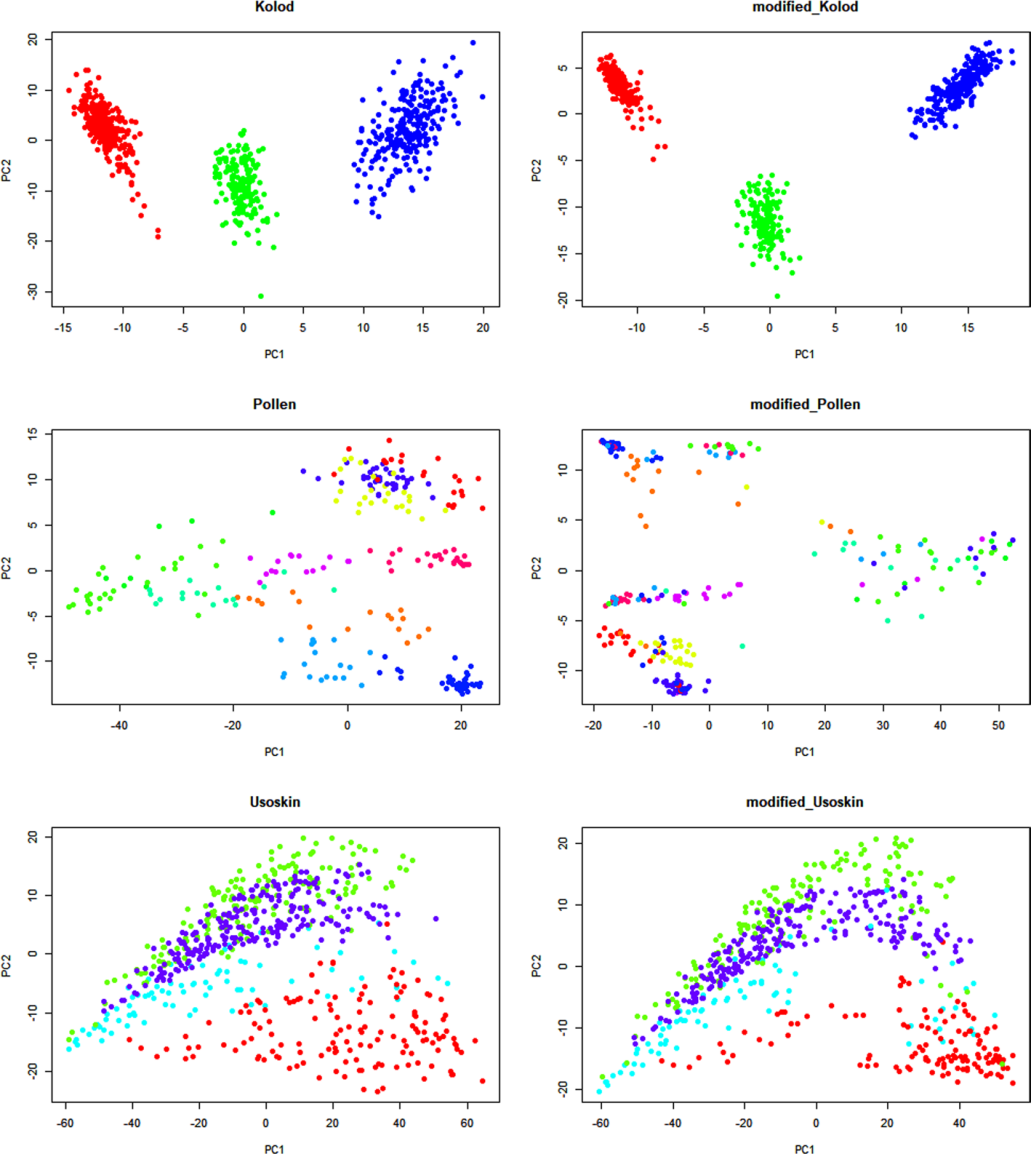
The PCA visualization of raw data and modified data. We performed SCC in three different datasets. The three datasets are Kolod, Pollen and Usoskin. Next, we performed principal component analysis on raw and new matrixs and used the first two principal components to draw the scatter plot. The left plots are the scatter plot of raw data and right plots are the scatter plot of modified data. As shown in Fig, we can draw a conclusion that SCC can make cells with the same types aggregate more closely

As shown in the Table 1, SCC reduce the distance of same type cells compared with raw data. Compared with other methods, SCC has the best performance in most scRNA-seq datasets except for Kolod dataset. The result shows that SCC can significantly make the cells with same type closer. We add one to the raw matrix and modified matrix and transform them by log. Finally, we perform principal component analysis on the new matrix. The first two principal components are used for visualization [10]. As shown in Fig. 4, we can clearly observe that SCC can reduce the distance of same type cells.

#### SCC can enhance the clustering of cell subpopulation

**Fig. 5:**
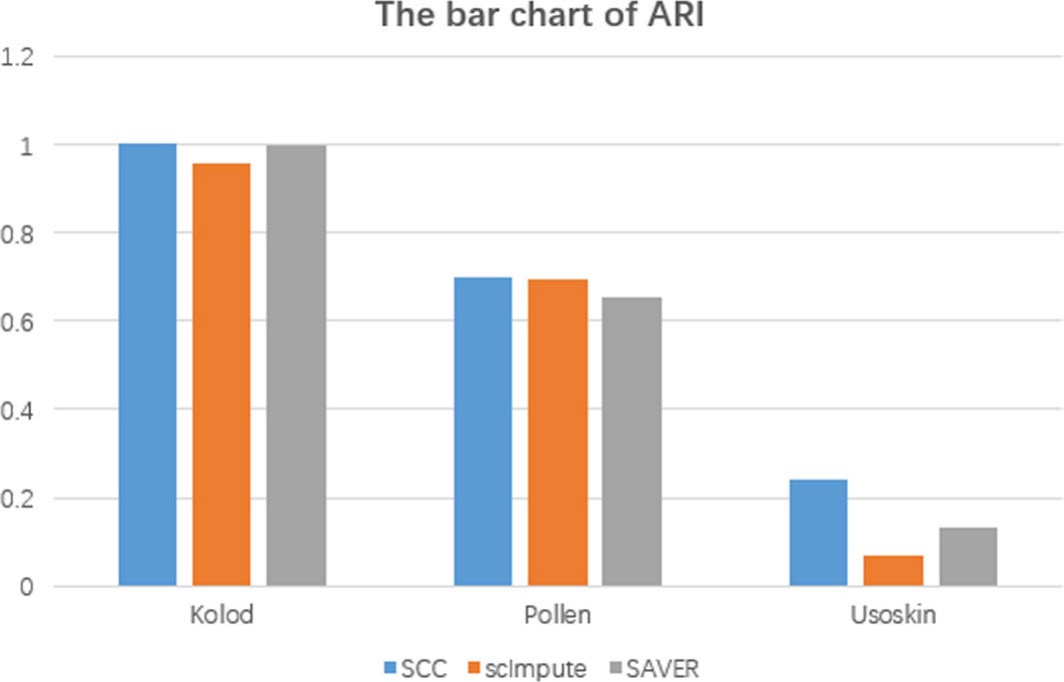
The barchart of ARI values. We run SCC, scImpute and SAVER in three different datasets (Kolod, Pollen and Usoskin) and performed K-means clustering in modified data. Finally, we calculated the ARI value and draw the bar chart of different methods

After the modification of gene expression, we further cluster the modified data. In the existing clustering algorithm, K-means clustering algorithm is a popular iterative solution-clustering algorithm [11]. We run SCC, scImpute and SAVER in three datasets and perform k-means clustering in the modified results. Finally, we calculate the ARI values in different methods. The result is shown in Fig. 5. The detailed values are shown in Table 2.

**Table 2:** Table of ARI value

	SCC	scImpute	SAVER
Kolod	1	0.957535	0.995985
Pollen	0.70043	0.694498	0.654509
Usoskin	0.24264	0.07003	0.131585

As shown in Table 2, the ARI values of SCC are obviously higher than other methods, especially in Kolod dataset, the ARI value of clustering is 1, which indicating that the two labels of clustering and real cell types are the same. From this table, we can draw a conclusion that SCC can significantly enhance the clustering of cell subpopulation in most datasets.

## Discussion

The primary limitation in scRNA-seq technology is high dropout noise level caused by the poor sensitivity of scRNA-seq technology, which makes low-expression genes hard to detect. scImpute and SAVER are existing tools for solving the noise in scRNA-seq data. They estimate the correct expression of genes by clustering similar cells and taking advantage of gene-to-gene relationships. However, they ignore cell-to-cell heterogeneity in the same types, which is important for cell heterogeneity. Compared with existing tools, SCC can retain the cell heterogeneity by modifying the expression of each cell by the nearest neighbor cells. Comprehensive expereiments show that SCC has a better performance compared with other tools in terms of intra-class distance of cells and the clustering of cell subpopulation.

## Conclusions

In conclusion, we proposed a method SCC (single-cell complementation) to resolve the noise (especially dropouts) in scRNA-seq data. SCC focuses on the gene expression that is largely affected by poor sensitivity of mRNA, while retaining the expression of genes with high expression level. The main idea of SCC is the complementation of similar cells. For each cell, we find the nearest neighbour cells by scmap and estimate the true expression value of dropouts by a mixture model. Compared with other methods, we can retain the cell heterogeneity by replacing clustering with detecting the nearest neighbor cells. We perform SCC and the other two methods in three different scRNA-seq datasets. The result shows that SCC can significantly reduce the intra-class distance of cells and enhance the clustering of cell subpopulation. Another advantage of SCC is memory-efficient (SCC only solves a cell at a time, we also can deal multiple cells at a time to improve the speed) and it can deal with tens of thousands of cells dataset on a laptop.

In the future, we will continue our research based on the previous scRNA-seq work. After the modification of gene expression, we will cluster the cells in the modified data. Some existing clustering methods (such as SC3) have a good performance in the clustering of scRNA-seq data [12]. We hope to find the shortcomings of the existing clustering methods and decide how to propose a better clustering methods for scRNA-seq data.

## Methods

**Fig. 6:**
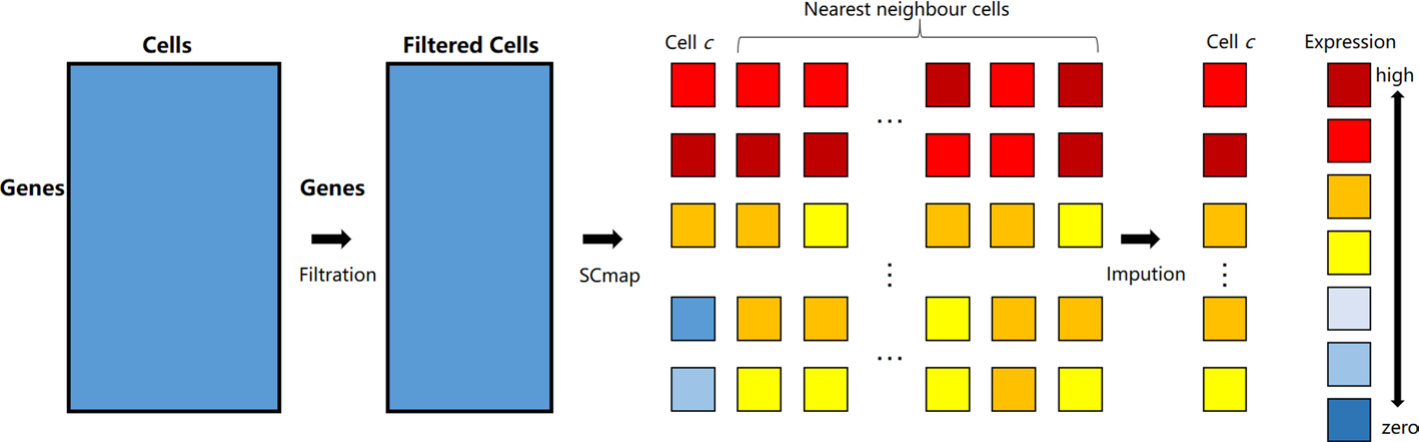
The flow chart of the core algorithm of SCC. The input of SCC is the matrix of scRNA-seq data and there are three main steps in SCC. The first step is the filtration of outliers. In second step, we obtain the nearest neighbor cells set of every cell *c* by SCmap. In third step, we solve the dropouts of the cell *c* according to the nearest neighbor cells

In order to solve the noise of scRNA-seq data, we had developed this new method, SCC, which can be used to recover the expression of genes with dropouts. The basic process is shown in Fig. 6, the core algorithm of SCC consists of three steps. The first step is the filtration of outliers as we recover the gene expression by the nearest cells, the outliers have a great impact on the modified result. The second step is the detection of nearest neighbor cells through another method scmap. The third step is the modification of gene expression. We propose a mixture model to describe the distribution of actual gene data and estimate the correct value of genes through EM algorithm. The detailed description of each step is introduced in the following sections.

### The filtration of outliers

The input of our method is the matrix $X_{g \times c}$ of scRNA-seq data and the g (row) represent genes and c (column) represent cells. Outliers are the result of technical limitations, which have a great influence on the cells clustering [8]. At first, we use principal component analysis (PCA) to reduce the dimension on matrix *X* and calculate the distance matrix on the data after dimension reduction. The matrix of scRNA-seq data is a sparse matrix and PCA can resolve the sparse problem and accelerate the speed of calculation [13]. We select the first two principal components to calculate the distance matrix $D_{c \times c}$.

The distance matrix $D_{c \times c}$ can be calculated based on the PCA-transformed data. For each cell *c*, we select the nearest cell and calculate the nearest distance $dis_{c}$. For the set of $dis_{c}$, we find the first quartile $dis_{q1}$ and third quartile $dis_{q3}$. For the cells that satisfy the follow formula, $dis_{c}>dis_{q1}+1.5(dis_{q3}-dis_{q1})$, we regard them as outlier cells [6]. We first delete the outlier cells as they have an bad effect on imputation and implement our experiment in the rest cells matrix $X_{g \times c1}$ (*c*1 represents the rest cells).

### The detection of nearest neighbour cells

In this step, we find the top twenty nearest neighbor cells of each cell by calculating the Euclidean distance first, but this step spends a lot of time in the calculation of distance. In fact, projecting individual cells onto most similar neighbor cells is also an important method to reduce the batch effect of scRNA-seq data. We detect nearest neighbor cells by another method SCmap rather than the Euclidean distance calculation. SCmap is a very convenient method to project a cell to the nearest neighbors, which use the cosine similarity, Pearson and Spearman correlations to calculate similarities [14]. An important feature of SCmap is very fast and it takes only about 1 min to select features (important genes) and calculate nearest neighbour cells for 40,000 cells. We choose SCmap to select 20 nearest neighbor cells $C=\{c_{1},c_{2},c_{3},\ldots ,c_{20}\}$ for each cell *c*. And then we filter lower similarity cells (lower similarity means that the similarity difference is greater than 0.1 ). The rest cells set $C=\{c_{1},c_{2},c_{3},\ldots ,c_{n}\}$ will be retained for recovering the gene expression profiles.

### The modification of gene expression

**Fig. 7:**
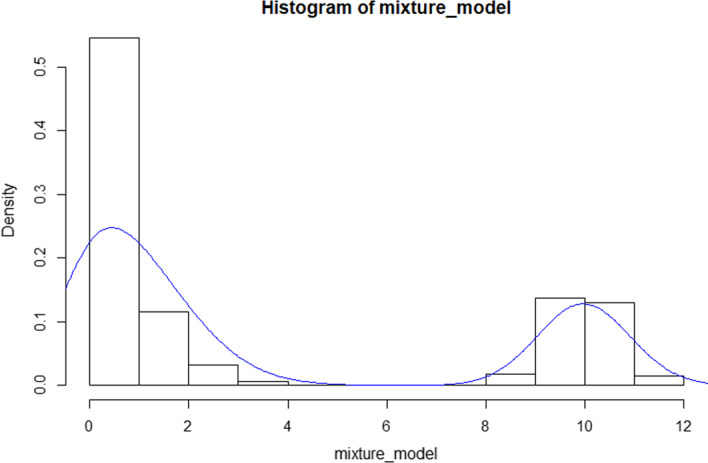
The distribution of mixture model. We used the mixture model to describe the distribution of scRNA-seq. It can be seen that the distribution of mixture has similar bimodality with the distribution of scRNA-seq. Therefore, the distribution of scRNA-seq can be described by the mixture model

After we obtain the nearest neighbour cells set $C=\{c_{1},c_{2},c_{3},\ldots ,c_{n}\}$ of each cell *c*, we need to predict the real gene expression value for each cell [15]. For the genes of each cell, we classify genes into three categories: high-expression, low-expression and zero-expression. The high-expression genes have a large amount of mRNA, so they are likely to be detected by scRNA-seq technology. The low-expression genes have less mRNA number, which leads to most dropouts in scRNA-seq data because of low detection sensitivity. Moreover, zero-expression means that the real value of gene expression is zero. For the different genes expression level, we construct a mixture model to determine whether a zero value is a real value or a dropout. Because the low-expression genes are difficult to detect, the most genes in scRNA-seq data tend to be bimodal expression distribution. Therefore, we describe actual data by a mixture model with three components. The first component is a normal distribution used to represent the high-expression genes, and the gene expression of high-expression genes in same cell types are different because of biological factors. The second component is a binomial distribution used to represent the actual low-expression genes data. For low-expression genes, the mRNA detection rate is set to *p*. The probability of each mRNA being detected is *p*, so low-expression genes scRNA-seq data can be described by binomial distribution. The third component is a zero distribution to represent genes expression value is zero. As shown in Fig. 7, the distribution of mixture model has similar bimodality with scRNA-seq data distribution. Therefore, the mixture model can describe the distribution of scRNA-seq data well. Most methods log the input matrix with a pseudo count 1 to deal with scRNA-seq data. In our method, the matrix $X_{g \times c1}$ is not transformed by log with a pseudo count, so it is reasonable to describe the actual distribution of scRNA-seq data for scRNA-seq data by the binomial distribution. Please note that scRNA-seq data does not represent the correct gene expression value, we first use a mixture model to describe actual scRNA-seq data distribution and then estimate the correct gene expression value.

For each cell *c*, we construct a different mixture models to modify the cell *c* gene expression. The different models have different proportions and parameters. For each gene *g*, its expression is modeled in cells set $C=\{c_{1},c_{2},c_{3},\ldots ,c_{n}\}$. The model formula is as follows (Eq. 2)

2 $$\begin{aligned}&f(x)= \lambda _{1} Normal (x,\mu , \sigma ) + \lambda _{2} Binomial (x,p,N) + \lambda _{3}0 \\&subject \quad to \quad \lambda _{1}+\lambda _{2}+\lambda _{3}=1,\quad x\in \{X_{g,c_{1}},X_{g,c_{2}},\ldots ,X_{g,c_{n}}\} \end{aligned}$$

The $\lambda _{1}, \lambda _{2}, \lambda _{3}$ are the probabilities of three distribution and the sum of $\lambda$ is one, the $\mu , \sigma$ are the mean and standard deviation of Normal distribution and the *p*, *N* is the detect probability and total mRNA number of every gene. We distinguish whether a gene is 0 expression or low expression or high expression through parameter $\lambda _{1}, \lambda _{2}, \lambda _{3}$. If the $\lambda _{1}$ or $\lambda _{2}$ of a gene is highest and $\lambda _{3}$ is very low, we think that the zero value in the scRNA-seq is likely a dropouts. On the other hand, if the $\lambda _{3}$ of a gene is highest, the real value of the gene is likely zero. $X_{g,c_{n}}$ is the value of gene *g* in cell $c_{n}$. We calculate every value of $\lambda$ and identify which $\lambda$ is highest. If $\lambda _{1}$ is highest, the gene is likely a high expression gene and the value of $X_{g,c}$ remains uncharged to retain the cell-to-cell heterogeneity. If the $\lambda _{2}$ is highest, we think the gene is likely a low expression gene which leads to most dropouts. And we replace the scRNA-seq data with the modified value $N*p$ (the expectation of the second model), the dropouts can be resolved. If the $\lambda _{3}$ is highest, the real value of $X_{g,c}$ is likely zero. We put the modified value into the matrix and get the modified gene expression data.

### The estimation of parameters

The advantage of this model is that it obtain the modified gene expression by neighbor cell complementation as it assumes the value of dropouts relates to nearest neighbor cells. The parameters in the model are estimated by Expectation–Maximization (EM) algorithm [16]. The expectation–maximization algorithm is to find the maximum likelihood estimation or the maximum posterior estimation of parameters in the probabilistic model, in which the probabilistic model depends on the hidden variables that cannot be observed. We first set an initial value for every parameter and then calculate every value’s probability in three components. The number of iteration is set to 100 and the iteration will stop when the difference of parameters is small (the threshold is set to 0.01).

The E step: The initial value of $\mu$ is the mean of the nearest neighbor cells and the initial value of $\sigma$ is the standard deviation. The initial value of *p* is 0.1 because the detection rate of mRNA is about 5–15%. The *N* is an integer number less than 10. All the $\lambda$ are set to one-third. For the gene *g* in nearest cells set $C=\{c_{1},c_{2},c_{3},\ldots ,c_{n}\}$, we calculate the probabilities of every gene expression in three components for modifying the parameters in M step.

The M step: We have obtained the probabilities $Pro_{n \times 3}$ of gene *g* in nearest cells set $C=\{c_{1},c_{2},c_{3},\ldots ,c_{n}\}$. Then we calculate the new probabilities $Pro_{n \times 3}=Pro_{n \times 3}/rowsum(Pro)$ that the values belong to three components. We set $P1=sum(Pro[,j])$, $P2=sum(Pro[,j]*matrix[g,])$. The mean of every component *j* will be calculated by $Mean_{j}=P2/P1$ and the value of $\lambda _{j}=P1/n$ (n is the number of nearest cells). We set $P3=sum(Pro[,j]*(matrix[g,]-Mean_{j})^2)$ and the standard deviation of every component *j* will be calculated by $Dev_{j}=sprt{P3/P1}$. After obtaining the values, we can calculate the parameters: $\mu$, $\sigma$, *N*, *p*, $\lambda$.

## Data Availability

The datasets and code is freely accessible at the website: https://github.com/nwpuzhengyan/SCC.

## Abbreviations

scRNA-seq: Single-cell RNA sequencing
SCC: scRNA-seq complementation
PCA: Principal component analysis
ARI: Adjusted Rand Index
